# Looking beyond the obvious: IgA nephropathy in a liver transplant recipient

**DOI:** 10.4103/0971-4065.65305

**Published:** 2010-04

**Authors:** P. M. Kar, D. Yi, S. M. Kar

**Affiliations:** 1507 S. Hiawassee Road, Suite # 102, Orlando, FL 32836, USA

**Keywords:** Cyclosporine, IgA nephropathy, post liver transplant, renal failure

## Abstract

Seventeen years after a liver transplant for biliary atresia, an adolescent presented with renal failure. The serum level of cyclosporine was sub-therapeutic; and, in spite of dosage adjustments, the patient’s status did not improve. Given the patient’s age, future renal transplant was a feasible consideration. However, this warranted investigation in the form of a renal biopsy in an attempt to confirm the underlying cause(s) of her renal failure. The renal biopsy revealed marked alteration in the renal anatomy due to IgA deposition, interstitial fibrosis, and hyaline arteriopathy.

## Introduction

IgA nephropathy is the commonest cause of primary (idiopathic) glomerulonephritis throughout most developed countries of the world.[1] While its initial description can be traced back approximately 40 years, IgA nephropathy remains incompletely understood to this day. Of the many proposed mechanisms that are believed to be responsible for the pathophysiology behind IgA nephropathy, only one has been linked to the liver and its role in IgA clearance from the circulatory system.[2] Indeed, there is a growing correlation between IgA nephropathy and the gastrointestinal system, specifically the liver.[3–6] This case report of IgA nephropathy in a post-liver-transplant patient not only adds further significance to the correlation between liver disease and IgA nephropathy, but also introduces the possibility of a direct correlation between IgA nephropathy and liver transplantation.

## Case Report

Our patient was an 18-year-old female with a past medical history of hypertension, biliary atresia status post-liver transplant at the age of 1 year and asthma who presented with complaints of fatigue, weakness, nausea, vomiting, and diarrhea lasting approximately three to four weeks. Her primary care physician instructed her to seek medical attention after outpatient labs were found to be significant for severe anemia. Her home medications include amlodopine, azathioprine, cyclosporine, prednisone, and an albuterol metered-dose inhaler.

The patient’s vital signs were significant for tachycardia. She was not in acute distress and was alert and oriented. Her physical examination was unremarkable except for scars from a previous surgery. Admission laboratory findings were significant for hemoglobin 53 g/dL; sodium 134 mEg/L; potassium 3.4 mEg/L; chloride 96 mmol/L; CO_2_ 21 mmol/L; creatinine 16.3 mg/dL; glucose 80 mg/dL; and calcium 8.1 mg/dL. Liver function assays and coagulation studies were within normal limits; urine human chorionic gonadotropin was negative. The patient’s cyclosporine at the time of admission was 23 ng/mL. As a result, her drug dosage was adjusted and repeat levels on the fifth day of admission were reported at 93 ng/mL. Urinalysis was significant for +3 protein and +2 bacteria but negative for blood. Her spot urine protein and creatinine ratio was in the nephrotic range. Urine cultures were negative. Renal ultrasound revealed increased echogenicity of the kidneys, measuring 8.8 cm on the left and 10.1 cm on the right side.

**Figure 1:**
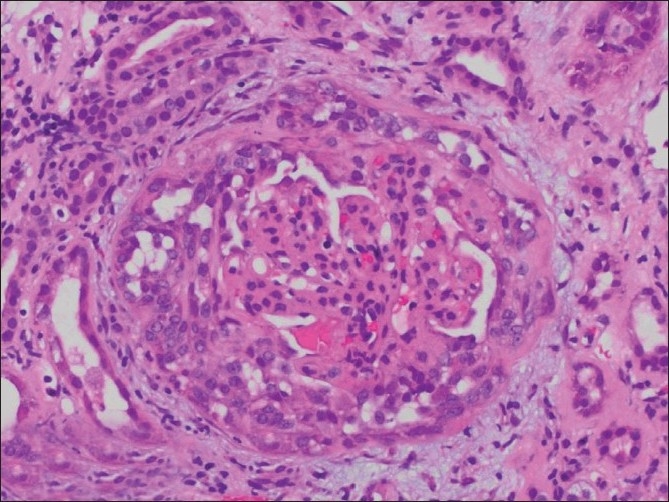
Light Microscopy showing focally variable mesangial, endocapillary, and epithelial proliferation (H and E, ×230)

**Figure 2:**
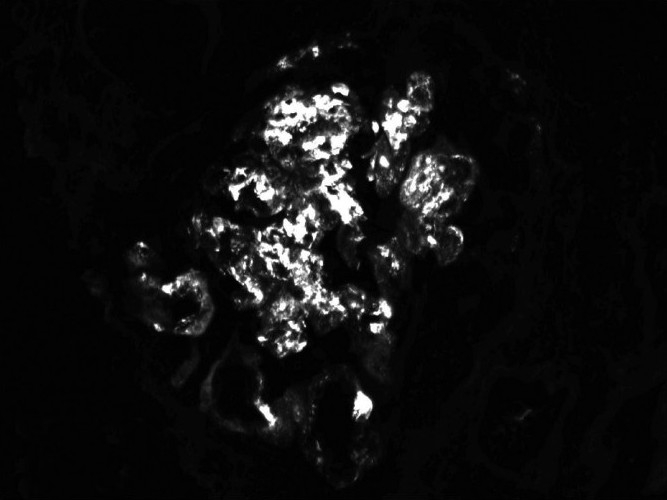
Direct immunofluorescence showing IgA: Coarsely granular mesangial and capillary (3+)

After appropriate transfusions, the patient was started on hemodialysis, and responded well to treatment. Renal biopsy was pursued, as the patient could be deemed a candidate for renal transplant, given her age. The biopsy [Figures 1 and 2] showed IgA nephropathy with crescent formation and acute and chronic calcineurin toxicity. The patient was eventually discharged in stable condition and remained dialysis-dependent.

## Discussion

The basic pathophysiology behind IgA nephropathy centers on deposition of IgA in the glomerular mesangium. This IgA is predominantly polymeric IgA1, which is mainly derived from the mucosal immune system.[7] This is clinically manifested by frequent presentations of IgA nephropathy involving gross hematuria following upper respiratory-tract infections or acute gastroenteritis. The relationship between the gastrointestinal system and IgA nephropathy is noteworthy, especially since the most common form of secondary IgA nephropathy is associated with chronic liver disease.[8–9] In this scenario, predisposition of IgA deposition in the kidneys is thought to be secondary to impaired removal of IgA-containing complexes by the Kupffer cells in the liver.[10]

Perhaps of greater intrigue is that IgA nephropathy has been implicated as a cause of renal insufficiency or failure in patients who are status post-liver transplantation.[11] Interestingly, these patients often take one or more immune-modulating agents, such as Prednisone, Cyclosporine, and Azathioprine; these medications have been studied as possible treatment options for IgA nephropathy.[12–14]

## Conclusions

Chronic kidney disease is a known and frequent complication of solid organ transplantation that is well-documented in the literature.[15–16] Our liver transplantation patient developed end-stage renal disease secondary to IgA nephropathy. This further strengthens the correlation between IgA nephropathy and diseases of the gastrointestinal tract but raises another consideration of possible etiologies of kidney disease in transplantation patients who present with end-stage renal disease other then chronic rejection or Cyclosporine toxicity. Although there is evidence of calcineurin toxicity in this case as indicated by interstitial fibrosis and hyaline arteriopathy, this case also highlights the potential pitfall in treating end-stage renal disease in a patient with immunosuppressive therapy: reflexively attributing the renal failure to the immunosuppressant without considering other etiologies.
